# Denitrifying bacterial communities in surface-flow constructed wetlands during different seasons: characteristics and relationships with environment factors

**DOI:** 10.1038/s41598-021-82438-3

**Published:** 2021-03-01

**Authors:** Jia-ming Wei, Li-juan Cui, Wei Li, Yun-mei Ping, Wan Li

**Affiliations:** 1 Beijing Construction Engineering Group Environmental Remediation Co. Ltd, Beijing, 100051 China; 2 National Engineering Laboratory for Site Remediation Technologies, Beijing, 100872 China; 3 Institute of Wetland Research, Chinese Academy of Forestry, Beijing, 100091 China; 4 The Beijing Key Laboratory of Wetland Ecological Function and Restoration, Beijing, 100091 China; 5 Beijing Hanshiqiao National Wetland Ecosystem Research Station, Beijing, 101399 China

**Keywords:** Ecology, Wetlands ecology

## Abstract

Denitrification is an important part of the nitrogen cycle and the key step to removal of nitrogen in surface-flow wetlands. In this study, we explored space–time analysis with high-throughput sequencing to elucidate the relationships between denitrifying bacteria community structures and environmental factors during different seasons. Our results showed that along the flow direction of different processing units, there were dynamic changes in physical and chemical indicators. The bacterial abundance indexes (ACEs) in May, August, and October were 686.8, 686.8, and 996.2, respectively, whereas the Shannon-Weiner indexes were 3.718, 4.303, and 4.432, respectively. Along the flow direction, the denitrifying bacterial abundance initially increased and then decreased subsequently during the same months, although diversity tended to increase. The abundance showed similar changes during the different months. Surface flow wetlands mainly contained the following denitrifying bacteria genus: unclassified Bacteria (37.12%), unclassified *Proteobacteria* (18.16%), *Dechloromonas* (16.21%), unranked environmental samples (12.51%), unclassified *Betaproteobacteria* (9.73%), unclassified *Rhodocyclaceae* (2.14%), and *Rhodanobacter* (1.51%). During different seasons, the same unit showed alternating changes, and during the same season, bacterial community structures were influenced by the second genus proportion in different processing units. ACEs were strongly correlated with temperature, dissolved oxygen, and pH. Bacterial diversity was strongly correlated with temperature, electrical conductivity, pH, and oxidation reduction potential. Denitrifying bacteria are greatly affected by environmental factors such as temperature and pH.

## Introduction

Bacteria in constructed wetland participate in the decomposition and transformation of pollutants, which is one of the key mechanisms to remove pollution. Some reports have shown that denitrifying bacteria account for 60–86% of total nitrogen removal^1^. Denitrifying microorganisms exhibit rich species diversity^2^, although in some Paleozoic fungus groups or specific fungi have some roles in denitrification^3^, denitrification is primarily a bacteria activity, and more than 50 species of bacteria have been shown to have denitrifying activities^4^. Therefore, it is important to study the denitrifying bacterial community structures of wetlands. Lee and Kang used high-throughput sequencing to identify denitrifying bacterial community structures at different soil depths^5^, and Wang et al.^6^ revealed the wetland community structures of autotrophic denitrification bacteria. Additionally, Cao et al.^7^ assessed the denitrifying community structures of natural wetlands and constructed wetlands, and Fu et al.^8^ discussed different carbon sources for constructed wetland plants and denitrifying community structures. Santoro et al.^9^ used nirS/K as molecular markers; the salinity/denitrifying nitrite concentration gradient of the coastal wetland aquifer can be used to identify microbial diversity, with unique microbial groups identified at a very low space scale (40 m distance). Recent studies have focused on different types of wetlands and vertical depth, as well as the denitrifying community structures under different environmental conditions for both natural wetlands and constructed wetland. Fu et al.^10^ found that aerobic denitrifiers were entirely dominant in the middle and upper layers of the constructed wetland, where obligate halophilic, aerobic denitrifiers Zobellella occurred. Zhang et al.^11^ found that the variations of bacterial community presented a significant seasonal change and Gillisia (belonging to Bacteroidetes) and Woeseia (affiliating with Gammaproteobacteria) were the two primary components in the rhizosphere soils.

At present, most studies focus on the community structure of denitrifying bacteria in constructed wetland and its response to a single environmental factor, but do not discuss the community structure and spatial distribution of denitrifying bacteria in constructed wetland in different seasons^1^. We used Miseq high-throughput sequencing to evaluate the denitrifying bacterial community structure during different seasons in different processing units through a space–time three-dimensional analysis using redundancy analysis (RDA). We also explored the relationships of these community structures with environmental factors in order to evaluate the surface flow wetland spatial distributions of denitrifying bacterial community structures.

## Methods

### Experimental site and sample design

**Figure 1:**
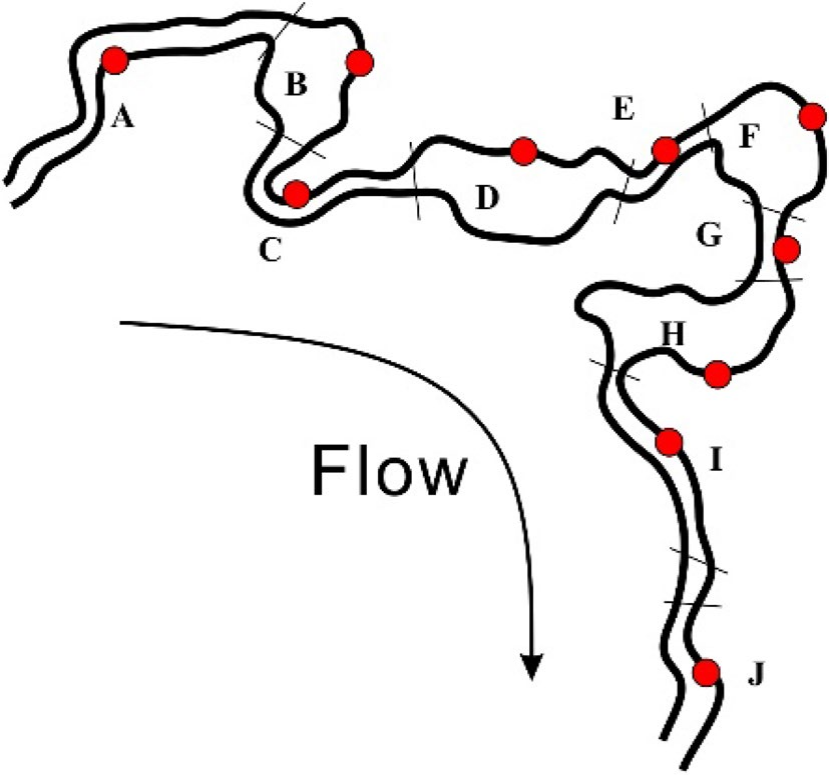
Sample locations at the surface-flow constructed wetland.

The study area was located in Shunyi district of Beijing, Beijing Wildlife Rescue and Breeding Center, a surface-flow wetland (6′14.40″40°N, 42′35.71″116°E). Because the Beijing Wildlife Rescue and Breeding Center was not open to tourists, the influence of artificial factors on the bacteria environment was minimized. Figure 1 shows the layout of the flow wetlands (A–J). The main species of each units were evaluated: *Typha orientalis*, *Eichhornia crassipes*, *Acorus calamus*, *Sagittaria sagittifolia*, *Eleocharis congesta*, *Nymphoides peltatum*, *Oenanthe javanica*, *Monochoria korsakowii*, *Sparganium stoloniferum*, and *Iris tectorum*. In August (summer), October 2015 (autumn), and May 2016 (spring), The surface litter was stripped during samping, and the sediments with the depth of 0–10 cm were sampled. Overlying water in the sediment around the site was collected in triplicate. Sediment samples were cryopreserved at − 80 °C until molecular biology analysis. For environmental factor analysis, water samples were stored at 4 °C.

### DNA extraction, polymerase chain reaction (PCR) amplification, and sequencing

DNA extraction was carried out using an Omega Soil DNA Kit (Omega Bio-tek, Norcross, GA, USA) using the following primers^12^: cd3aF, 5′-GTSAACGTSAAGGARACSGG-3′,R3cd, 5′-GASTTCGGRTGSGTCTTGA-3′. PCR was carried out using TransGen AP221-02, with TransStart Fastpfu DNA polymerase in a 20-μL reaction system containing 5 × FastPfu buffer (4 μL), 2.5 mM dNTPs (2 μL), forward primer (5 μM; 0.8 μL), reverse primer (5 μM; 0.8 μL), FastPfu polymerase (0.4 μL), template DNA (10 ng), and ddH_2_O to 20 μL. PCR was carried out with an ABI GeneAmp 9700 instrument using the following parameters: 95 °C for 3 min; 27 cycles of 95 °C for 30 s, 55 °C for 30 s, 72 °C for 45 s; and 72 °C for 10 min. All samples were assessed using AxyPrepDNA gel recovery kits (Axygen Biosciences, Union City, CA, USA), and PCR products were eluted with Tris HCl solution and detected by 2% agarose gel electrophoresis. Quantitative PCR was carried out using a QuantiFluor-ST blue fluorescence system (Promega, Madison, WI, USA). Using bridge PCR and reversible end analysis^13^, in combination with the Illumina MiSeq platform and standard methods for high-throughput sequencing, we obtained data from each round of PCR and then analyzed the template DNA sequences.

### Water physicochemical properties

Dissolved oxygen (DO), salinity, oxidation reduction potential (ORP), pH, electrical conductivity (SpCond), total dissolved solids (TDSs), temperature, and other indicators of water quality were analyzed using a portable multiparameter YSI-exobiology instrument (YSI, USA). Determination of total nitrogen (TN) was carried out using a SMARTCHEM200 automatic chemical analyzer (WestCo, USA). Total organic carbon (TOC) using the determination of total organic carbon analyzer (Elementar, Germany).

### Statistical analysis

Statistical analysis of the community composition of each sample was carried out using the Qiime platform and RDP Classifier^14^ Bayesian algorithm based on a 97% similarity level for operational taxonomic units (OTUs) in representative sequence taxonomical analysis^15^ and SILVA databases. If the taxonomic databases in some taxonomic lineages had no scientific name for class, the tag “norank” was used. Additionally, the classification was marked as “unclassified” in the classification score at a particular level was low.

R language was used to generate all optimized sequence maps of OTUs using the obtained sequences for OTUs with similarities of more than 97%. OTUs with similarity levels of 97% or more were further analyzed using R language^16^, the ACE index (estimated OTU number) in the community, Shannon-Weiner index (H′; a bacterial diversity index; larger values indicate higher community diversity).

Differences among denitrifying bacterial communities were evaluated using R language, and correlations among water index parameters and bacterial community structures were assessed using Pearson correlation analysis. R language with nonmetric multidimensional scaling analysis and principal component analysis (PCA) were used to evaluate environmental factors. Canoco 5 with redundancy analysis (RDA) was used to assess water factors and the relationships between the denitrifying bacterial community and aquatic environment.

## Results and discussion

### Physicochemical properties of water

Physicochemical properties of water and associated environmental factors according to the flow directions of surface flow wetlands are shown in Table 1. Analysis of variance for indexes with *p* values of less than 0.05 showed that all indexes exhibited large variations during different months.

**Table 1:** Physicochemical characteristics of the surface-flow constructed wetlands in each unit.

Wetland location	DO/mg·L^−1^			ORP/mV			pH			Salinity/ng·L^−1^			SpCond/mS·cm^−1^			TDS/g·L^−1^			Temp/°C			TN/mg·L^−1^			TOC/mg·L^−1^		
	5	8	10	5	8	10	5	8	10	5	8	10	5	8	10	5	8	10	5	8	10	5	8	10	5	8	10
A	2.281	3.917	2.417	− 59.000	− 57.800	− 144.633	7.673	10.223	7.717	0.313	0.277	0.227	0.640	0.574	0.469	0.416	0.367	0.300	16.053	28.075	16.835	1.956	1.600	2.398	5.525	6.996	5.683
B	2.644	3.983	1.810	− 134.000	− 55.667	− 261.833	7.530	10.173	7.650	0.300	0.270	0.220	0.614	0.573	0.455	0.399	0.367	0.291	16.103	28.099	17.444	4.572	1.586	1.687	7.353	8.715	5.133
C	3.085	4.040	0.963	− 57.667	− 54.300	− 270.000	7.553	10.133	7.610	0.290	0.270	0.227	0.597	0.572	0.464	0.388	0.366	0.297	16.193	28.207	16.007	2.669	1.282	3.420	5.358	8.277	5.430
D	2.921	4.083	1.003	− 21.333	− 53.433	− 236.300	7.897	10.107	7.693	0.273	0.270	0.230	0.561	0.571	0.467	0.365	0.366	0.299	19.073	28.206	15.373	2.281	0.822	2.902	6.937	4.592	6.237
E	2.366	4.130	0.853	− 47.667	− 52.633	− 206.300	7.883	10.077	7.677	0.267	0.270	0.230	0.550	0.570	0.467	0.358	0.365	0.299	16.710	28.375	15.411	2.301	0.810	2.070	7.322	4.540	6.969
F	1.601	4.170	1.140	− 30.333	− 52.033	− 186.967	7.657	10.050	7.637	0.283	0.270	0.230	0.582	0.569	0.466	0.378	0.364	0.298	16.583	28.245	15.695	1.310	0.546	1.017	7.090	4.684	5.426
G	1.321	4.203	1.490	− 8.667	− 51.600	− 218.667	7.557	10.030	7.553	0.290	0.270	0.230	0.595	0.567	0.467	0.387	0.363	0.299	17.273	28.372	15.781	0.949	0.583	0.894	6.989	4.665	4.226
H	1.095	4.240	2.307	− 19.000	− 51.300	− 206.267	7.477	10.017	7.490	0.280	0.270	0.227	0.577	0.565	0.466	0.375	0.362	0.298	18.097	28.290	15.697	1.124	0.519	0.808	7.536	7.166	4.280
I	1.168	4.273	1.697	− 21.667	− 51.100	− 211.733	7.437	10.003	7.573	0.283	0.270	0.230	0.579	0.564	0.466	0.377	0.361	0.298	19.010	28.439	15.741	1.222	0.540	0.690	8.724	7.578	5.323
J	1.891	4.300	2.540	− 79.333	− 50.933	− 208.467	7.297	9.990	7.620	0.317	0.270	0.227	0.644	0.563	0.465	0.419	0.360	0.297	15.167	28.291	14.901	1.623	0.595	0.752	9.036	8.349	6.099

(5: May; 8: August; 10: October).

Some indexes exhibited large variations during different months according to the flow directions of surface flow wetlands. For example, DO was first reduced and then increased in May, but increased in August and showed differences compared with that in May and October. The salinity was first reduced and then increased in May but then remained stable from August to October. Some indexes showed similar changes according to the flow directions of surface flow wetlands. For example, ORP showed an initial decrease followed by an increase. At the same time, pH, SpCond, TDSs, and TN showed reduced variability over time. The changes in temperature were minimal, although the temperature was higher in the summer and autumn than in the spring.

Surface flow wetlands are in direct contact with the environment and are greatly influenced by outside environmental factors^17^. Thus, most physicochemical factors of the water samples showed large variability.

### Denitrifying bacteria diversity and abundance

**Figure 2:**
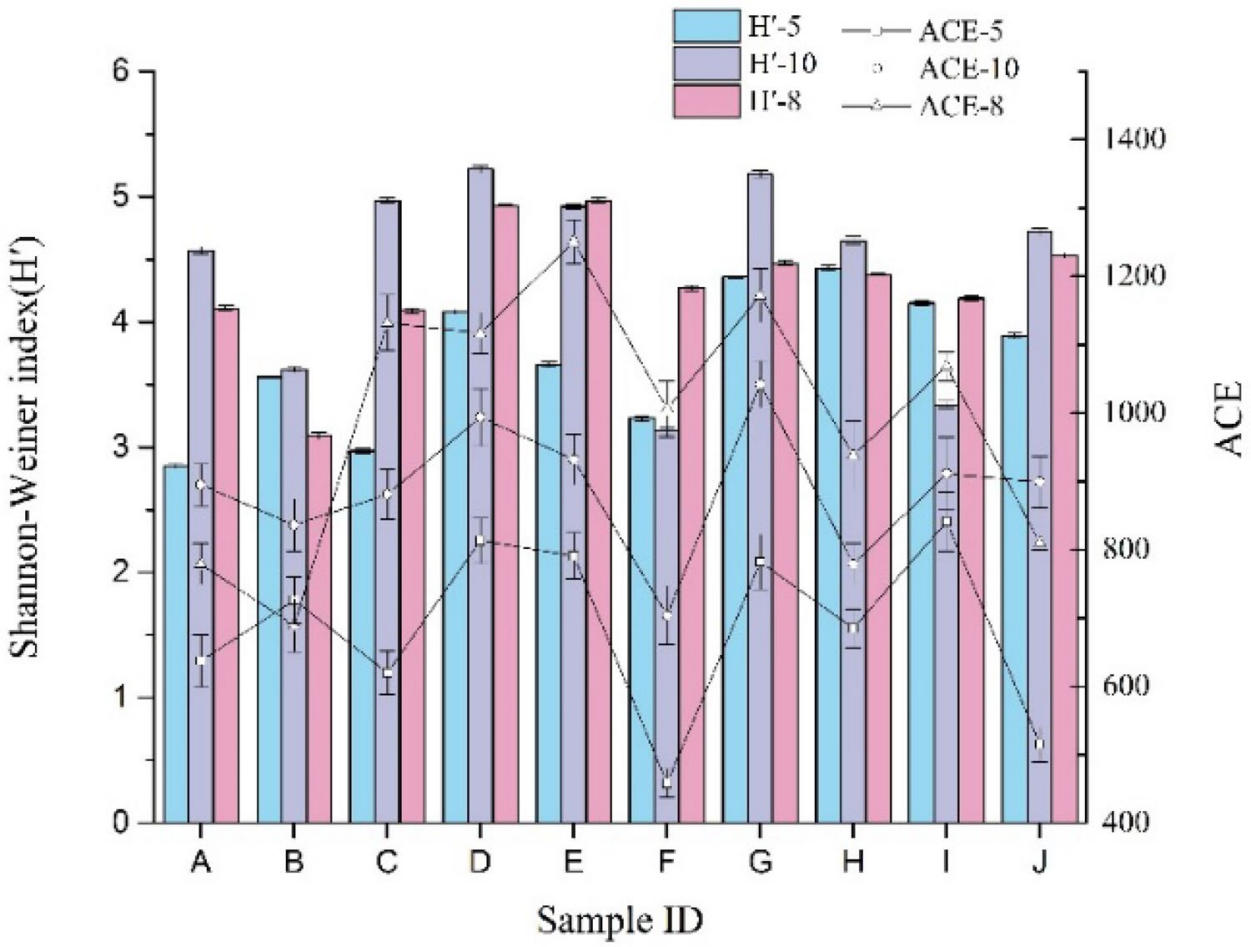
Biodiversity and abundance of the surface-flow constructed wetland in each unit.

For 10 samples from different seasons showing 97% similarity in clustering analysis, the numbers of OTUs differed in May, August, and October (575, 869, and 741, respectively), and the Fig. 2 showed that the denitrifying bacterial abundance indexes (ACEs) were 686.8, 996.2, and 887.3 in May, August, and October, respectively. Additionally, the Shannon-Weiner indexes (H′) were 3.718, 4.303, and 4.432, respectively, indicating that the abundance tended to increase initially, followed by a decrease, and diversity tended to increase. The different seasons affected both the denitrifying bacteria abundance and diversity. Abundance was the largest in August, but its diversity was lower than that in October. These data suggested that the main species became dominant during August, affecting the structure of the denitrifying bacteria.

For different processing units, the abundance and diversity of denitrifying bacteria varied slightly; both the ACE and H′ index showed low variability. The units F, H, and J showed greater declines than the initial values. In May, the ACE index peaked, with a value of 841 at location I. In August, the ACE index peaked at location E (1251), and that in October peaked at location G (1042). In different months, denitrifying bacteria abundances showed similar changes. Because bacterial diversity in the flowing water and static water were affected by different factors, the surface flow wetlands will be susceptible to various factors, and the bacterial community interactions with internal and external environmental factors will be important for bacterial survival^18^. Additionally, the number of constructed wetland bacteria decreases as the depth and distance increases^19,^^20^, suggesting that denitrifying bacteria may be affected by physical and chemical indicators of changes in water.

### Community structure of denitrifying bacteria

**Figure 3:**
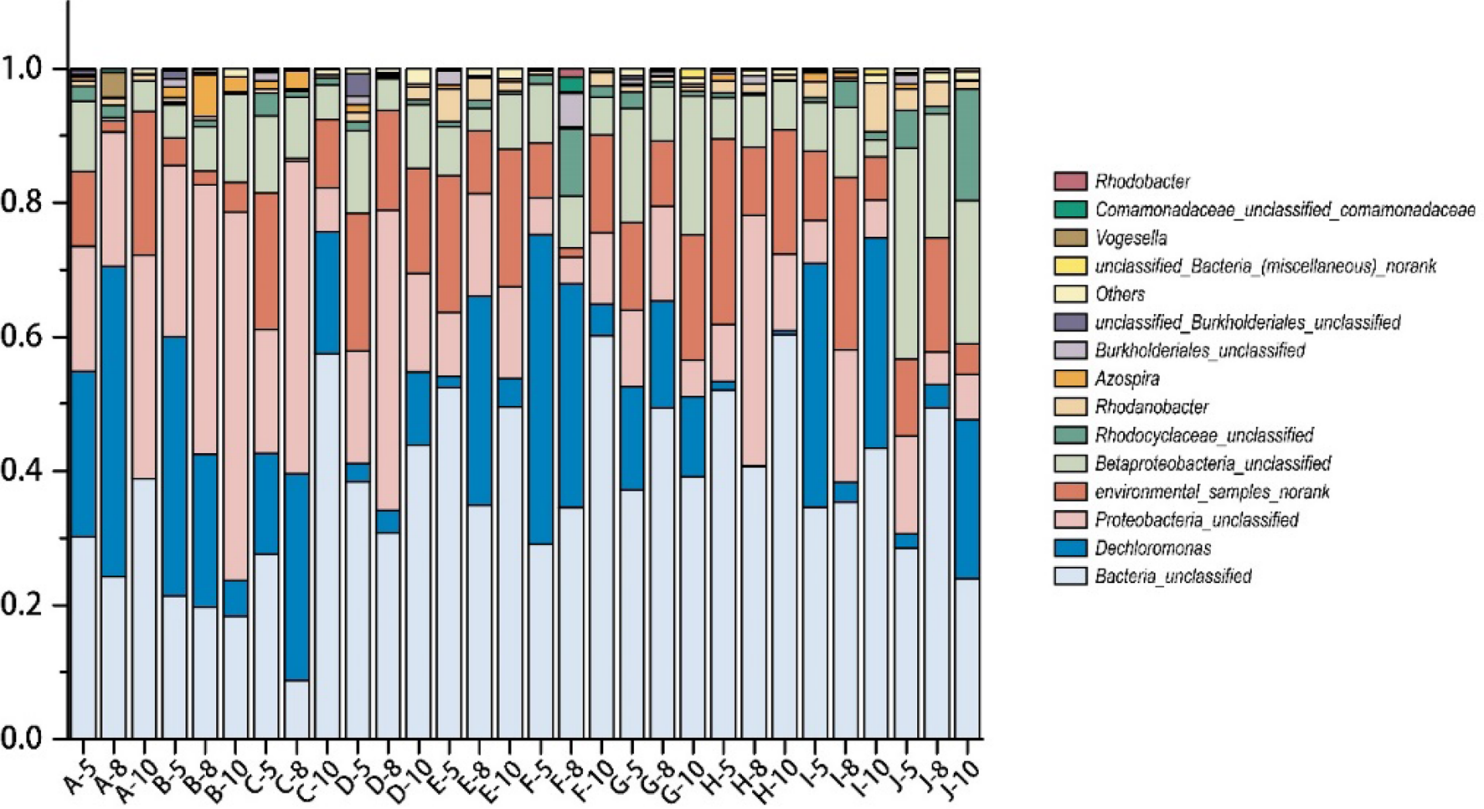
Community structure of the surface-flow constructed wetland in each unit.

Similar OTUs (97% similarity) were identified by sequencing. Database analysis of sequence alignment results revealed that there were many bacteria in the environmental samples that could not be cultivated but that showed high similarity; thus, the denitrifying bacteria were mostly present in the surface flow wetlands and were not cultured. Figure 3 shows statistical analysis of the denitrifying bacterial categories in a histogram format. During the different months, OTUs mainly belonged to seven genera: unclassified bacteria (37.12%), unclassified *Proteobacteria* (18.16%), *Dechloromonas* (16.21%), unranked environmental samples (12.51%), unclassified *Betaproteobacteria* (9.73%), unclassified *Rhodocyclaceae* (2.14%), *Rhodanobacter* (1.51%), and other genera (2.62%, representing less than 1% each). Several genera have also been found in surface flow wetlands^21^ and other types of constructed wetlands^22–24^, albeit with different proportions.

The same processing units showed different denitrifying bacterial community structures during different seasons and were always changing. Unclassified bacteria showed a greater weight during May for the A processing unit, although its weight was lower than that of *Dechloromonas* in August. In October, unclassified bacteria had become the most dominant group, and the proportion of *Dechloromonas* was extremely low. For the B processing unit, from May to October, the proportion of *Dechloromonas* was decreased, and the proportion of unclassified *Proteobacteria* was increased, overtaking *Dechloromonas*. For the C and D processing units, unclassified *Proteobacteria* were dominant in May, and unclassified bacteria were dominant in August and October. For the E, G, H, I, and J processing units, unclassified bacteria were dominant at all time points. For the F processing unit, the bacterial groups were similar to those of the B processing unit, with proportion of *Dechloromonas* decreasing and the proportion of unclassified bacteria increasing in October.

**Figure 4:**
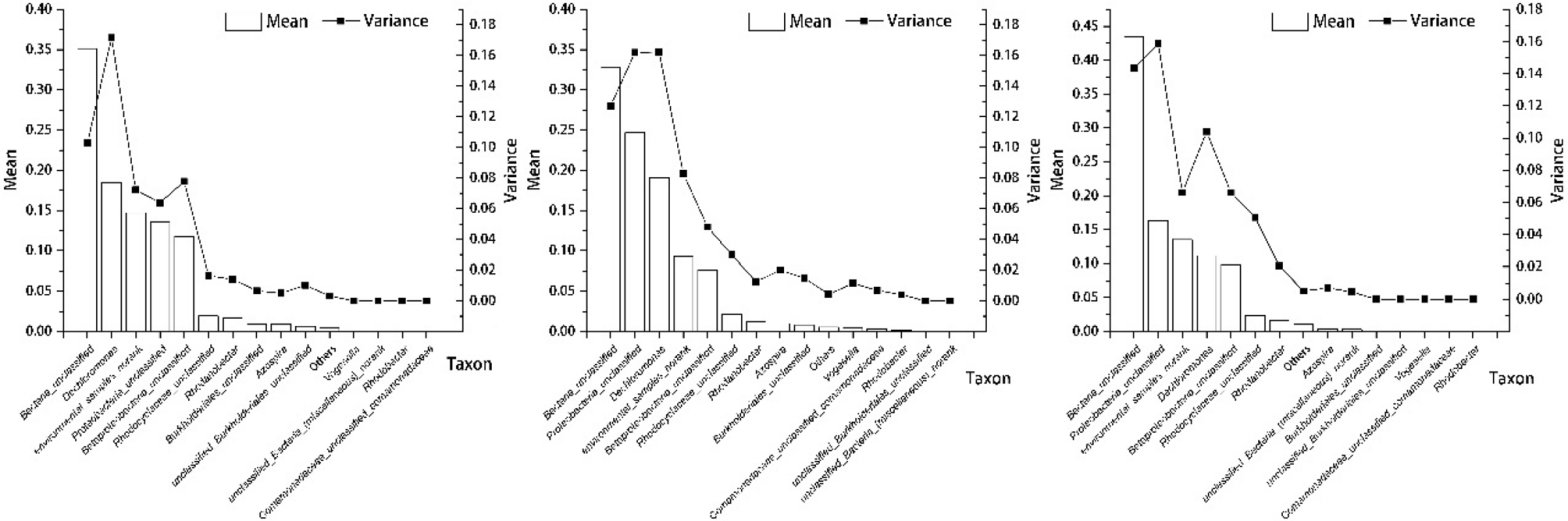
Mean and variance of different denitrifying bacterial taxa in the surface-flow constructed wetland (May, August, and October).

Figure 4 shows the means and variances of denitrifying bacteria genus proportions among different processing units and seasons. The means and variances of the dominant genus were large at the same time during different seasons. Thus, the dominant genus often determined the changes in denitrifying bacteria community structures during different seasons in the same unit. However, the greatest variance was observed in the genus *Dechloromonas*, which was the second most dominant genus in May. This suggested that this genus showed greater changes in different processing units than others. In August, the largest variances were observed in the genus *Dechloromonas* and in unclassified *Proteobacteria*, which had lower means than unclassified bacteria. Similar results were observed in October. The largest variance was observed in unclassified *Proteobacteria*, indicating that the denitrifying bacterial community structures were affected by the second dominant genus over time in the different processing units.

**Figure 5:**
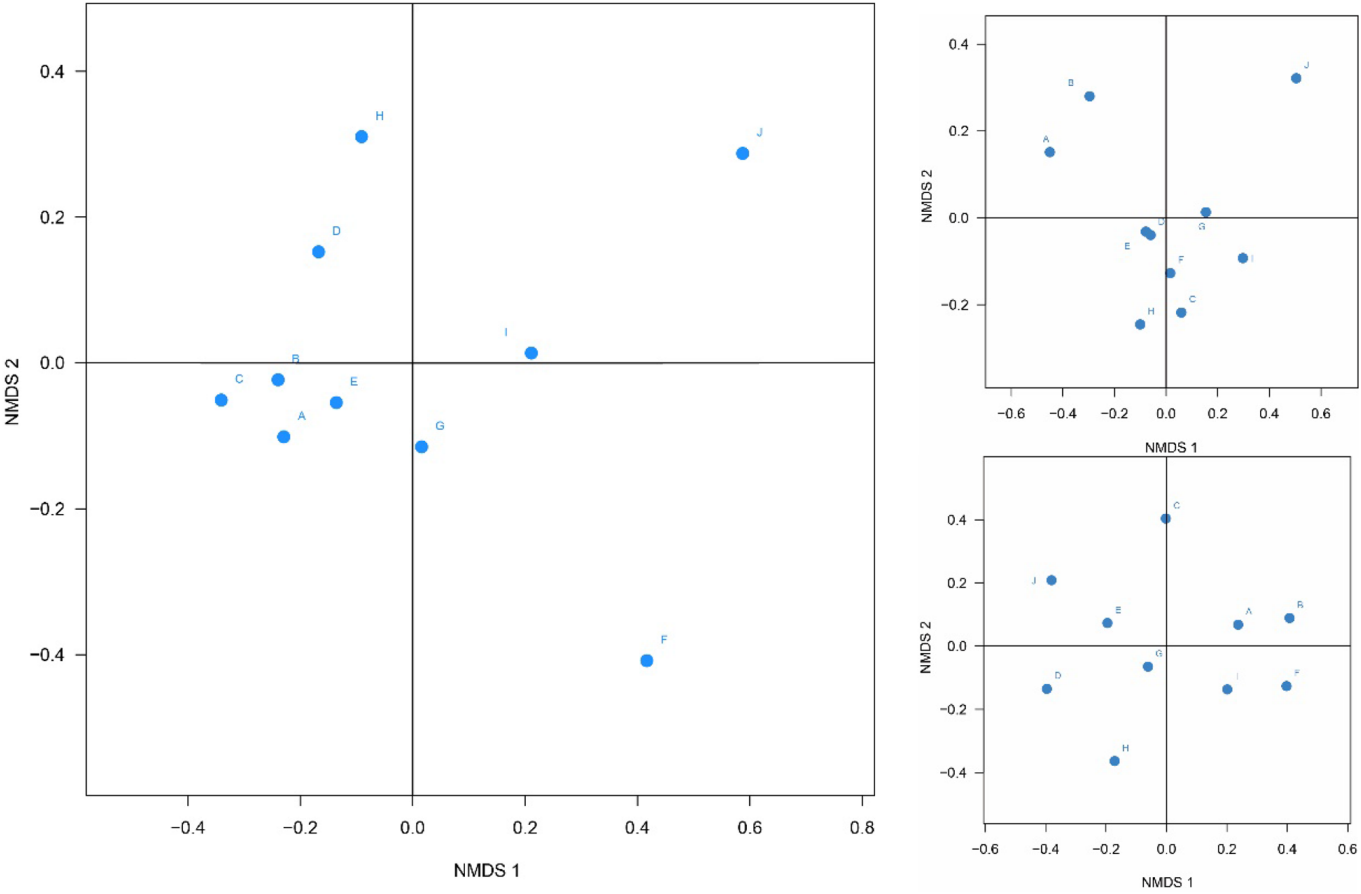
Nonmetric multidimensional scaling map (May, August, and October).

For the denitrifying bacteria community structures in different months, we used nonmetric multidimensional scaling to determine the similarities between different processing units during the same months. As shown in Fig. 5, in May, A, B, C, and E showed high similarity, whereas other samples were more dispersed. The distances between D and H and between G and I were shorter than the other distances. F and J were alone in a group. Sample distributions were concentrated in August; C, D, E, F, G, H, and I were relatively similar, and D and E showed maximum similarity. A and B showed some similarity. In contrast, J was distinct. In October, distributions were more dispersed, and the distances between two points were not relatively similar, whereas the differences between the various processing units were higher.

### Relationships between denitrifying bacteria and environmental factors

**Figure 6:**
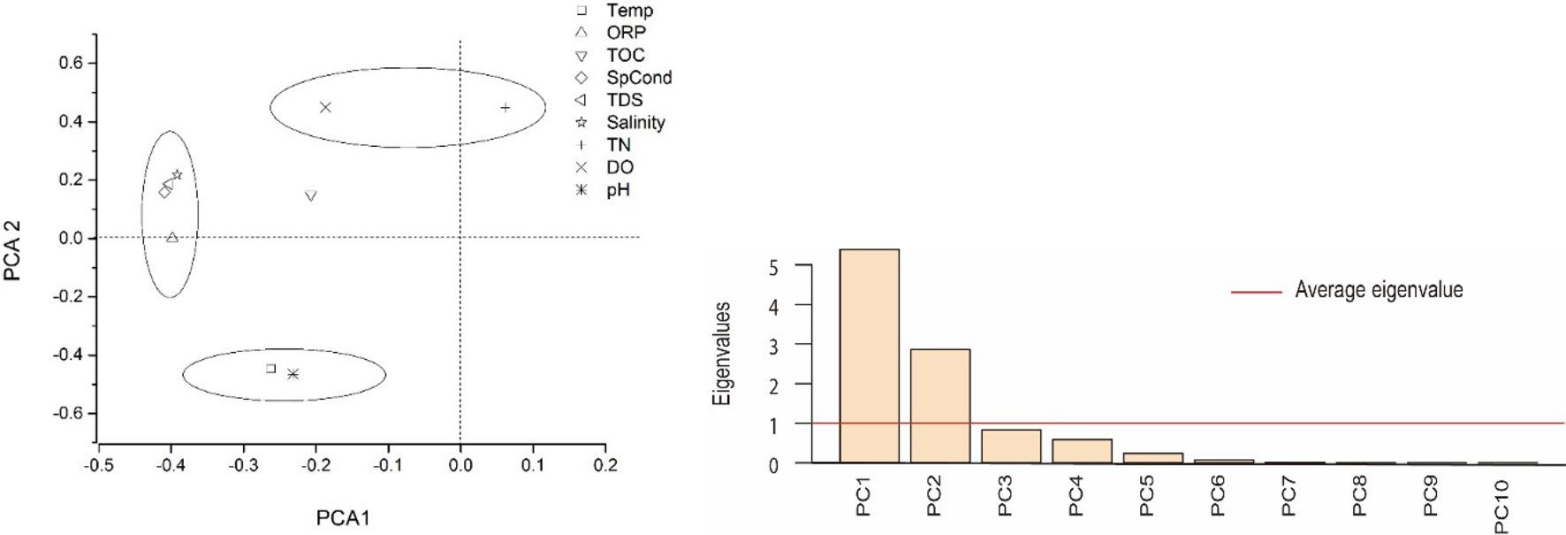
PCA of various environmental factors in the surface-flow constructed wetlands.

Next, we carried out PCA analysis to determine the main factors affecting denitrifying bacteria. After maximum variance orthogonal rotating (*p* = 0.05), there were two principal component eigenvalues that were greater than the average. The two top principal components contributed to 53.9% and 28.7% of the variance. The first principal component mainly reflected SpCond, TDSs, ORP, and salinity (factor loading was 0.409, 0.403, 0.398, and 0.403, respectively), and the second principal component reflected DO, TN, pH, and temperature (factor loading was 0.449, 0.449, 0.465, and 0.446, respectively). The load distribution characteristics of different environmental factors showed that the surface flow wetlands were affected by the main environmental factors, including temperature, SpCond, DO, pH, ORP, and TN (Fig. 6).

Table 2 shows bacterial abundance indexes and H′ index for different external environmental factors, as analyzed by Pearson correlation analysis. The results showed that the bacterial abundance was strongly correlated with temperature, DO, and pH, and H′ was strongly correlated with all parameters except TN.

**Table 2:** Relationships between biodiversity and environment factors.

	Temp	SpCond	DO	pH	ORP	TN
ACE	0.502**	− 0.301	− 0.507**	0.526**	− 0.136	− 0.328
H′	0.659**	0.869**	0.375*	0.570**	0.924**	− 0.221

**Significance at the level of 0.01; *Significance at the level of 0.05.

**Figure 7:**
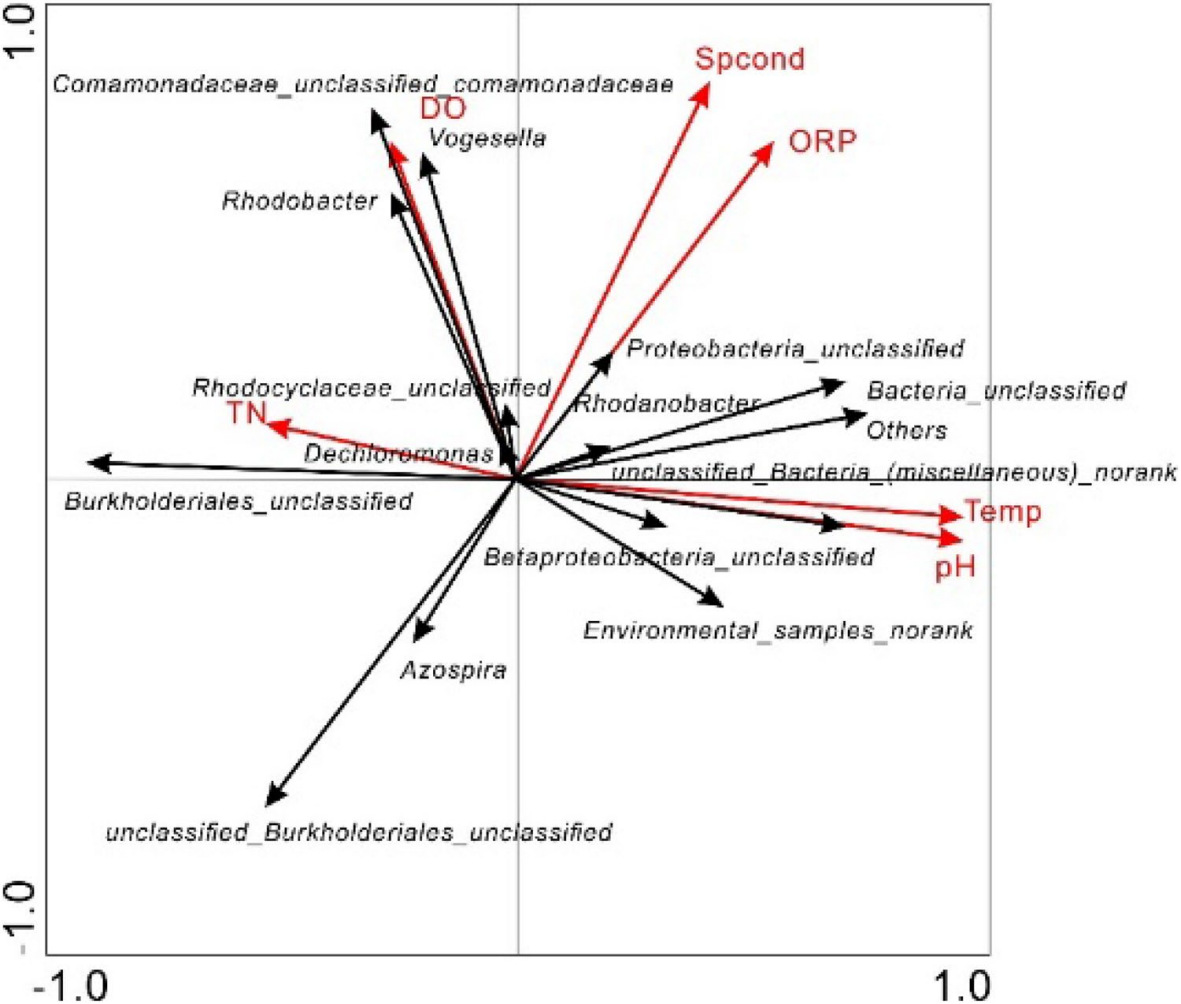
Relationships between denitrifying bacterial community structures and environment factors.

RDA was performed (Fig. 7) for analysis of community distributions and the relationships among environmental factors. For screening of the physicochemical factors of water and the proportions of denitrifying bacterial genera, standardization to center (Monte Carlo permutation) tests were used, and refinement of the information extracted from the first and second axes showed that the total explained variance rate was 80.94%. The results showed that all denitrifying bacterial genera were greatly affected by environmental factors, including temperature and pH, and that the effects of SpCond and ORP were similar. The predominance of unclassified bacteria and unclassified *Proteobacter* could be explained by positive correlations with temperature, pH, ORP, and SpCond and negative correlations with TN and DO. *Dechloromonas* showed the opposite trends. In contrast, unranked environmental samples were similar to unclassified *Betaproteobacteria*, with positive correlations for temperature and pH but negative correlations for TN and DO.

Denitrifying bacterial diversity is affected by water nutrient elements and other environmental factors. Most denitrifying bacteria were heterotrophic bacteria. In this study, the autotrophic denitrifying bacteria *Dechloromonas* accounted for a large proportion in each processing unit^25,26^,these bacteria can accumulate phosphate and exhibit denitrification activity, partly explaining the lack of TOC removal in association with the observed TN removal. The SpCond of the water reflected its salinity and could be explained by positive correlations with a high proportion of unclassified *Proteobacteria*. However, SpCond was not generally correlated with denitrifying bacterial abundance. Our results showed that the water SpCond in surface-flow constructed wetlands affected salinity-related denitrifying bacteria but did not affect other denitrifying bacteria. The ORP was positively correlated with denitrifying bacterial genera that were suitable for survival in a strong oxidizing environment, such as unclassified *Proteobacteria*.

Different physical and chemical properties can influence the structure of the bacterial community owing to the influence of different species on the living environment^27,28^. In this study, we assessed environmental factors that differed according to season and showed that denitrifying bacteria varied according to some environmental parameters. A comprehensive analysis of the trend of physical and chemical properties of water showed that all parameters except DO and salinity were not highly affected by season and that the trend of the abundances of denitrifying bacteria communities did not change with season along the flow direction of different processing units. However, environmental indicators have a more significant impact on different denitrifying bacteria, which also changes the diversity of denitrifying bacteria community. Accordingly, these results, combined with prediction models of the effects of environmental factors on nitrogen and phosphorus^29–31^, could be used to predict changes in the denitrifying bacterial community structure.

## Conclusions

In this study, we evaluated changes in denitrifying bacteria community structures with variations in environmental and water physicochemical factors. Our results showed that most of the physicochemical factors of water have similar trends in different seasons along the flow direction of different processing units. The denitrifying bacteria community structure was greatly influenced by season, but the variations in abundance were similar in different seasons. The same processing units showed different dominant denitrifying bacteria during different seasons, i.e., changes in variations and denitrifying bacteria diversity of communities. The denitrifying bacterial community structures were affected by the second dominant genus over time in the different processing units. The denitrifying bacterial abundance was also correlated with temperature, DO, and pH, and denitrifying bacterial diversity was correlated with temperature, SpCond, pH, and ORP. These finding provide important insights into the diversity and stability of denitrifying bacterial wetland communities.
